# A Ternary Age-Mixing Model to Explain Contaminant Occurrence in a Deep Supply Well

**DOI:** 10.1111/gwat.12170

**Published:** 2014-03-05

**Authors:** Bryant C Jurgens, Laura M Bexfield, Sandra M Eberts

**Affiliations:** 2 U.S. Geological Survey5338 Montgomery Blvd. NE, Suite 400, Albuquerque, NM 87109; 3 U.S. Geological Survey6480 Doubletree Ave., Columbus, OH 43229

## Abstract

The age distribution of water from a public-supply well in a deep alluvial aquifer was estimated and used to help explain arsenic variability in the water. The age distribution was computed using a ternary mixing model that combines three lumped parameter models of advection-dispersion transport of environmental tracers, which represent relatively recent recharge (post-1950s) containing volatile organic compounds (VOCs), old intermediate depth groundwater (about 6500 years) that was free of drinking-water contaminants, and very old, deep groundwater (more than 21,000 years) containing arsenic above the USEPA maximum contaminant level of 10 µg/L. The ternary mixing model was calibrated to tritium, chloroflorocarbon-113, and carbon-14 (^14^C) concentrations that were measured in water samples collected on multiple occasions. Variability in atmospheric ^14^C over the past 50,000 years was accounted for in the interpretation of ^14^C as a tracer. Calibrated ternary models indicate the fraction of deep, very old groundwater entering the well varies substantially throughout the year and was highest following long periods of nonoperation or infrequent operation, which occured during the winter season when water demand was low. The fraction of young water entering the well was about 11% during the summer when pumping peaked to meet water demand and about 3% to 6% during the winter months. This paper demonstrates how collection of multiple tracers can be used in combination with simplified models of fluid flow to estimate the age distribution and thus fraction of contaminated groundwater reaching a supply well under different pumping conditions.

## Introduction

The groundwater-age mixture in water from a public-supply well (PSW) is the most important characteristic to determine when assessing the intrinsic susceptibility of the water to contamination. It determines how fast a contaminant can reach the well, how high concentrations might be and how long concentrations might remain elevated after remediation of the contaminant source. Because PSWs are commonly constructed to yield the largest amount of potable water, screened intervals are typically long, spanning several meters to hundreds of meters of aquifer. This is particularly common in deep alluvial aquifers in the southwest United States. PSWs with long screens in the semiarid to arid Southwest typically produce water with a wide range of groundwater ages, spanning decades to several millennia (Reichard et al. 2003; Plummer et al. 2004a, 2004b; Kulongoski et al. 2005; Manning et al. 2005; Jurgens et al. 2010).

The distribution of groundwater ages in water produced by a PSW can be determined using particle-tracking methods coupled to detailed numerical 3D groundwater flow models or using inverse modeling of tracer concentrations with lumped parameter models (LPMs; Maloszewski and Zuber 1982; Weissmann et al. 2002; Cornaton and Perrochet 2006a, 2006b; Paschke et al. 2007; Burow et al. 2008; Green et al. 2010). Recently, Eberts et al. (2012) showed that LPMs can give age distributions that are similar to those predicted by particle-tracking methods for wells with water of mixed age based on similar conceptual models and calibrated to similar tracer data. Previous work has also demonstrated the utility and use of LPMs to develop multimodal mixing models of tracers to understand the age distribution of groundwater in wells (Katz et al. 2004; Long and Putnam 2006; Corcho Alvarado et al. 2007; Solomon et al. 2010; Stolp et al. 2010).

Although LPMs have been widely used to interpret tracer concentrations for understanding groundwater age, carbon-14 (^14^C) has not been used frequently alongside tracers of recent recharge to understand groundwater-age mixtures spanning thousands of years. When ^14^C data have been used with LPMs, the atmospheric input of ^14^C over the past 50,000 years generally has been held constant, assuming the atmospheric input has been steady at 100 percent modern carbon (pmC), even though concentrations in the atmosphere have fluctuated as a result of natural variations in the earth's geomagnetic field (Stuiver 1961, 1965) and, more recently, from aboveground nuclear weapons testing (Clark and Fritz 1997; Hua and Barbetti 2004). In this study, a transient history of atmospheric ^14^C levels spanning the last 50,000 years was used to compute simulated ^14^C concentrations for comparison to measured concentrations during model calibration. The use of this variable input history with the LPMs provides correspondence to single, apparent ^14^C ages determined for water samples from radiocarbon programs such as CALIB, while enabling the entire age distribution—including young fractions of water containing tracers such as tritium (^3^H) and chlorofluorocarbons (CFCs)—to be estimated.

Wells with broad age distributions can contain contaminants that are associated with one or more fractions of the age distribution. For example, in the alluvial aquifer of the Middle Rio Grande Basin (MRGB), New Mexico, volatile organic compounds (VOCs) are commonly associated with recently recharged young groundwater, whereas elevated arsenic is commonly found in deep, very old groundwater (more than 18,000 years; Bexfield and Plummer 2003; Plummer et al. 2004a; Bexfield et al. 2012). PSWs within the basin near the city of Albuquerque sometimes produce water with arsenic concentrations above the USEPA maximum contaminant level (MCL) of 10 µg/L (effective in 2006). Thus, in the Albuquerque area, it was necessary to collect tracers of groundwater age that can characterize fractions of young and old groundwater containing different drinking-water contaminants. In some cases, the tracer data from a PSW alone is insufficient to adequately estimate the full groundwater-age distribution for a well and information about tracer concentrations at monitoring wells located at different depths nearby the well are needed to more accurately constrain the age distribution in water from the PSW. It also may be necessary to collect tracers at multiple depths within a pumping PSW to understand the age of groundwater entering the well.

Recently, the U.S. Geological Survey National Water-Quality Assessment (NAWQA) program's TANC topical study (Eberts et al. 2005) investigated the vulnerability of a public-supply well in Albuquerque, New Mexico (Bexfield et al. 2012). That study used concentrations of ^3^H, chlorofluorocarbon-113 (CFC-113), and ^14^C to develop binary mixing models between young and old components of groundwater for PSWs in the study area. Bexfield et al. (2012) showed that groundwater at intermediate depths (27 to 80 m below the water table) near a PSW contained as much as 45% young groundwater (recharged since about 1950) and trichloroethylene (TCE) at concentrations greater than 2 µg/L even though ^14^C concentrations indicated the groundwater was predominantly much older than 1000 years.

The fraction of old groundwater in the binary mixing model of Bexfield et al. (2012) was recognized as being bimodal and comprised of two distinct groundwater masses having different mean ages and distributions. However, the old fraction was treated as a single source of water represented by a broad distribution of ages because a primary focus of that paper was in obtaining estimates of the young fraction in groundwater. Because arsenic concentrations exceed the USEPA MCL in the oldest fraction, the concentration of arsenic in samples collected from the wellhead can only be completely understood by separating the two fractions of old groundwater entering the well and developing a ternary model of the resulting mixture. A more complete representation of the age distribution of the old fraction would account for the two primary components of old groundwater entering the well. This distinction leads to a ternary mixture with relatively younger old water, ranging from 6000 to 10,000 years old entering the well at intermediate depths combined with a very old component of groundwater, greater than 18,000 years old, entering the well at depths greater than 80 m below the water table.

In this study, a three component mixture of groundwater age was developed to describe tracer concentrations and provide a plausible age distribution for a PSW in Albuquerque, New Mexico. Although binary mixtures of young (less than 60 years) and old (more than 1000 years) groundwater have been developed in recent research (Corcho Alvarado et al. 2007; Solomon et al. 2010), ternary mixing models are more difficult to develop and calibrate because the data required to identify and constrain a ternary model is not easily obtained. For this study, the ternary model could be identified because of multiple tracer data collected from nearby monitoring wells as well as from inside the pumping well at different depths. In addition, a flow profile of the pumping well was used to segregate distinct bodies of water and their proportions of flow contributed to the well. The ternary mixing model was then used to describe arsenic concentrations from the PSW, which varied according to the time of year the well is sampled.

## Description of Study Area

**Figure 1:**
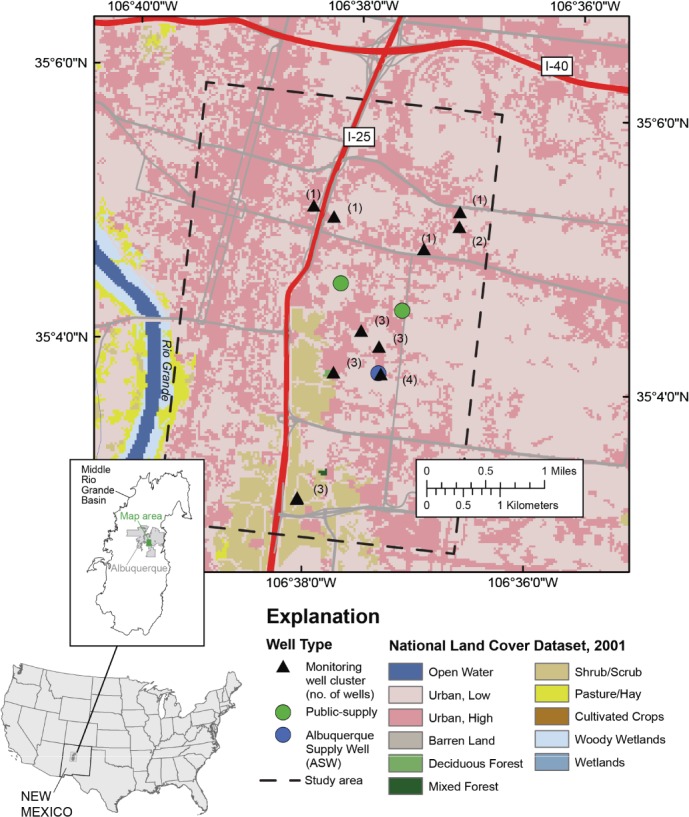
Map of land cover features of the Middle Rio Grande Basin (MRGB), New Mexico, and location of the study area in Albuquerque.

The study area lies in the MRGB and is an urban area located within the City of Albuquerque, New Mexico (Figure 1). The study area covers 24 km^2^, extending from the Rio Grande inner valley along its western boundary (minimum elevation about 1505 m) onto upland areas along its eastern boundary (maximum elevation about 1625 m). The climate is semiarid and the mean annual precipitation during 1914 to 2010 at Albuquerque was 22.1 cm (Western Regional Climate Center 2010). The 7922-km^2^ MRGB trends generally north to south and contains alluvial fill up to about 4500 m thick. The basin is bounded by mountains primarily on the north and east, and by a fault zone and adjacent structural basin on the west. The alluvial fill of the basin is composed primarily of the unconsolidated to moderately consolidated Santa Fe Group deposits of late Oligocene to middle Pleistocene age that were deposited in fluvial, lacustrine, or piedmont-slope environments. These deposits, in combination with hydraulically connected post-Santa Fe Group valley and basin-fill deposits of Pleistocene to Holocene age, form the Santa Fe Group Aquifer system of the basin (Thorn et al. 1993). Conditions within the aquifer system are generally unconfined, but they are semiconfined at depth. Depths to water in the general vicinity of Albuquerque range from about one meter to more than 200 m.

Recharge to the aquifer system of the MRGB is primarily through seepage from the Rio Grande and associated irrigation canals, although mountain-front recharge processes, subsurface groundwater inflow, and urban sources including leakage from water distribution systems also contribute water to the aquifer (Plummer et al. 2004a; Bexfield et al. 2012). Direct infiltration of precipitation across the landscape contributes little or no recharge. Groundwater discharges from the aquifer system include agricultural drains, groundwater withdrawals for public supply, and riparian evapotranspiration. Until the Albuquerque Bernalillo County Water Utility Authority began diverting surface water from the Rio Grande in 2008, essentially all drinking water for residents of the Albuquerque metropolitan area (population 713,000 in 2000; U.S. Census Bureau 2001) was supplied by groundwater withdrawals from the Santa Fe Group Aquifer system. As a result, large and extensive declines in water levels substantially altered the direction of groundwater flow in the area from being generally north to south to being toward the major pumping centers from all directions, including along east-west flowpaths from the Rio Grande (Bexfield and Anderholm 2002). The Albuquerque Bernalillo County Water Utility Authority plans to continue withdrawing groundwater to supplement supplies during drought and during times of peak demand in the summer (Albuquerque Bernalillo County Water Utility Authority 2007).

PSWs in the vicinity of Albuquerque generally range in depth from about 150 m to nearly 550 m and have screened intervals exceeding 150 m in length. Previous investigations have shown that groundwater produced by these wells is predominantly thousands of years old, but can include a small fraction that recharged within the past 50 years (Plummer et al. 2004a, 2004b; Bexfield et al. 2012). The primary groundwater contaminant of concern is arsenic, which naturally occurs in the aquifer sediments. Concentrations above the drinking-water standard of 10 µg/L (U.S. Environmental Protection Agency 2010a) typically are associated with older and (or) deeper groundwater. Investigations by Bexfield and Plummer (2003) and by Plummer et al. (2004a) found that sources of elevated concentrations of arsenic in groundwater of the MRGB included inflow of high-arsenic groundwater related to silicic volcanism in the Jemez Mountains north of the basin, and upwelling of mineralized water of deep origin along major structural features across the basin. Contaminants of anthropogenic origin also are of concern. In particular, VOCs have been detected in several PSWs in the Albuquerque area, generally at concentrations far below drinking-water standards (Plummer et al. 2008; Bexfield et al. 2012), but occasionally at concentrations that have required closure of a well located near a contamination site (U.S. Environmental Protection Agency 2010b, 2010c). Sites of known contaminant releases affecting the subsurface within the study area include current or former manufacturing, dry-cleaning, and transportation facilities with leaky storage tanks or formerly improper storage and (or) disposal practices (U.S. Environmental Protection Agency 2010b, 2010c; S. Arfman, New Mexico Environment Department, Petroleum Storage Tank Bureau, written communication, 2009).

## Methods

### Collection and Analysis

The Albuquerque study well (ASW) is typical of PSWs in the Albuquerque area, with a screen interval from 107 to 359 meters below land surface (mbls). The ASW withdraws water at a rate of about 11,660 L/min (3080 gallons per minute [gpm]) and the static water level at the well was about 76 mbls during 2007 to 2009. Water samples were collected from the ASW during four sampling events from June 2007 to May 2009. For each event, samples were analyzed for the age-dating tracers ^3^H, CFCs, and ^14^C as well as major ions, trace elements, and field parameters such as pH, dissolved oxygen (DO), and specific conductance (SC; Bexfield et al. 2012). When the ASW was sampled in June 2007 and November 2008, a single set of water samples was collected at the wellhead. In December 2007, a temporary submersible pump was installed in the ASW and water samples were collected at the wellhead and at several depths within the wellbore under pumping conditions, as described in more detail below. In addition, six sample sets were collected at the wellhead over a 64-h period in May 2009 to look at short-term changes in water-quality.

Groundwater samples were collected in accordance with procedures described in Koterba et al. (1995) and in the USGS National Field Manual (U.S. Geological Survey, variously dated). With the exception of depth-dependent (DD) samples, samples from the ASW were collected using the dedicated turbine pump; sample lines made of polytetrafluoroethylene (PTFE, hereafter referred to as Teflon) and nylon were attached to an existing tap located on the discharge line from the wellhead, prior to any water treatment. Water samples were collected after field measurements of water temperature, SC, pH, DO, and turbidity had stabilized.

^3^H samples were analyzed at the University of Miami Tritium Laboratory in Miami, Florida and ^3^H was determined by electrolytic enrichment and gas counting as described by Östlund (1987). Samples for CFCs were analyzed at the USGS Chlorofluorocarbon Laboratory in Reston, Virginia and CFC concentrations were determined by purge-and-trap gas-chromatography and electron capture detection (Bullister 1984; Bullister and Weiss 1988; Busenberg and Plummer 1992).

^14^C was determined by accelerator mass spectrometry through a contract with the University of Waterloo, Waterloo, Ontario, Canada (Drimmie et al. 1994) and by the National Ocean Sciences Accelerator Mass Spectrometry (NOSAMS) Facility at Woods Hole Oceanographic Institute (WHOI), Massachusetts (NOSAMS Facility 2010) and by Rafter Radiocarbon Laboratory (2010) in Lower Hutt, New Zealand. ^14^C concentrations were reported from the laboratory in percent modern. By radiocarbon convention (Stuiver and Pollach 1977), these laboratory values were normalized for carbon-13 fractionation to a standard δ^13^C of −25 per mil (VPDB). In order to calculate ^14^C ages correctly, measured ^14^C concentrations were converted to nonnormalized values using the δ^13^C of the sample, and the concentration reported in percent modern was converted to percent modern carbon (pmC) values (Stuiver and Pollach 1977; Mook and Van der Plicht 1999).

The ^14^C content of groundwater can be diluted by ^14^C-free sources involving geochemical reactions of carbon in the subsurface, which will affect groundwater ages estimated on the basis of ^14^C data. Bexfield et al. (2012) evaluated the ^14^C content of groundwater in the study area for dilution from ^14^C-free carbon sources using the geochemical inverse modeling programs PHREEQC version 2.17.1 (Parkhurst and Appelo 1999) and NetpathXL version 1.2 (Plummer et al. 1994; Parkhurst and Charlton 2008) and found the major reaction affecting measured ^14^C content in groundwater was dilution from ^14^C-free or “dead” organic carbon caused by long-term reduction of DO. This type of dilution normally is accounted for when estimating groundwater age by adjusting the initial ^14^C concentration, which is typically assumed to be a constant 100 pmC. However, the concentration of ^14^C in the atmosphere has been lower, and more often much higher, than 100 pmC over the last 50,000 years, mainly as a result of natural variations in the Earth's geomagnetic field (Stuiver 1961, 1965) and more recently from aboveground nuclear weapons testing (Clark and Fritz 1997). In order to use ^14^C as a tracer of old groundwater with LPMs, the measured ^14^C content, rather than the initial concentration, needs to be adjusted because the measured concentration is compared to the decayed transient history. In this study, the measured ^14^C concentration in samples from the PSW was adjusted using the corrected initial concentration given by NetpathXL and the following relation:
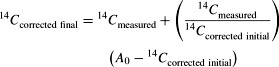
(1) where *A*_0_ is the assumed initial ^14^C content of water, usually 100 pmC.

For this study, *A*_0_ was assumed to be equal to the atmospheric ^14^C activity in precipitation, which was based on work by Plummer et al. (2004a). A more detailed discussion of the collection procedures, analysis of chemical constituents and tracers, and quality assurance of the data was given in Bexfield et al. (2012).

### ^3^H, CFC-113, and ^14^C Input History

**Figure 2:**
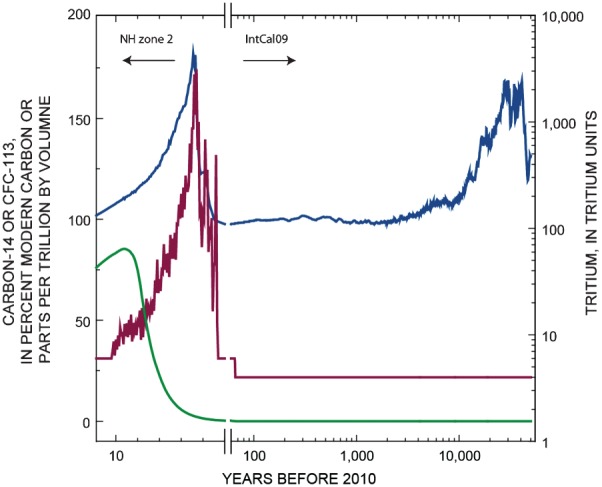
Carbon-14, chlorofluorocarbon-113, and tritium, concentrations in the atmosphere used to interpret the age of groundwater near Albuquerque, New Mexico. Carbon-14 activity younger than 58 years is from tropospheric carbon-14 bomb data for the northern hemisphere zone 2, NH zone 2 (Hua and Barbetti 2004). Carbon-14 activity older than 58 years is from the 2009 international calibration curve, IntCal09 (Reimer et al. 2009).

^3^H concentrations in precipitation from 1953 to 2002 for Albuquerque, New Mexico were obtained from an updated dataset of Michel (1989) and averaged into half-year increments (Figure 2). ^3^H concentrations were extrapolated for the period 2002 through 2010 using an exponential trend because ^3^H concentrations have been steadily declining since the 1980s. ^3^H concentrations in precipitation prior to aboveground nuclear weapons testing were assumed to be about 4 tritium units (TU). Northern Hemisphere atmospheric mixing ratios of CFC-113 were obtained from the U.S. Geological Survey Chlorofluorocarbon Laboratory (2009, http://water.usgs.gov/lab/).

In order to develop realistic models for mixtures of old, naturally-formed ^14^C and bomb-derived ^14^C, a ^14^C input curve was generated by combining the 2009 international radiocarbon calibration curve, IntCal09 (Reimer et al. 2009) with tropospheric ^14^C data for the northern hemisphere (zone 2; Hua and Barbetti 2004). Because ^14^C data were not collected for the period of 1950 to 1955 and 2000 to 2010, tropospheric ^14^C data were extrapolated using an exponential least squares fit of data compiled within 5 years of missing period (Figure 2). Use of this curve allows ages determined from a piston-flow LPM to correspond to piston-flow ages determined with radiocarbon calibration programs, such as CALIB (Stuiver and Reimer 1993). However, the transient input curve can be used with other LPMs that account for mixing within the aquifer and well bore and provide a more realistic estimate for the complete age mixture in the groundwater samples.

### Wellbore Flow

**Figure 3:**
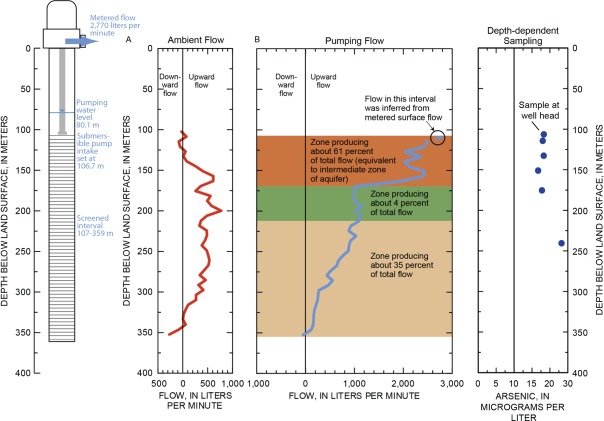
Wellbore flow and arsenic concentrations collected during DD sampling.

Electromagnetic flowmeter (EMFM) data were collected in the ASW under both ambient (nonpumping) and pumping conditions in order to determine the depths at which groundwater was entering or leaving the well screen and at what rate (Figure 3). The EMFM was used because the dedicated turbine pump in the ASW did not provide enough clearance for inserting dye-tracing or down-hole sampling equipment. To use the EMFM and complete the water-quality sampling, the dedicated turbine pump was removed and a submersible pump of smaller diameter was temporarily installed in the well for wellbore flow measurements under pumping conditions. The intake of the submersible pump was set at the top of the screened interval, about 6.1 m above the typical setting of the turbine pump intake. The submersible pump produced about 2460 to 2840 L/min (650 to 750 gpm), which was substantially less than the typical production of about 11,660 L/min (3080 gpm) by using the turbine pump. Although the total flow rate during the collection of DD flow and chemistry data under pumping conditions was lower than the rate during typical operation of the ASW, the results of the sampling effort are believed to provide useful information about relative flow and groundwater chemistry from different depths of the aquifer. Flow direction and velocity were measured at 6.1-m intervals throughout the length of screened interval (except below about 352 m in depth because of debris collection toward the bottom of the well). Turbulence at the bottom of the well likely affected the direction and magnitude of measurements from depths below about 340 m and were not considered reliable. The EMFM also recorded temperature and fluid resistivity (G. Stanton, U.S. Geological Survey, written communication, 2007).

On the basis of the flow profile under pumping conditions, five depths were selected for collecting DD groundwater samples in order to investigate changes in chemistry across depth intervals at which fairly large flow increases were observed. Because nearly 60% of the flow came from about the upper 68.6 m of the well screen, four of the five DD samples were collected across this interval. The samples were collected from about 115, 133, 151, 176, and 241 mbls using a submersible Bennett pump. Each of these samples represents the chemistry of all groundwater entering the screened interval below the depth of sampling; the groundwater was assumed to be thoroughly mixed. A groundwater sample also was collected at the wellhead (by using typical procedures for sampling of PSWs) to characterize the chemistry of water entering the well across its entire screened interval.

### Mixing Model Calculations

Mixing models were calculated for four samples from the PSW using TracerLPM (Jurgens et al. 2012). Only one sample, 09TS4, was modeled from the set of samples collected from the ASW in May 2009 because of the similarity in ^3^H and ^14^C concentrations among samples in the set and because the CFC-113 concentration was similar to the June 2007 CFC-113 concentration.

The concentration of an age tracer or arsenic concentration from the ASW was calculated using the following ternary mixing model equation:
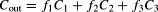
(2) where *C*_1_ is the concentration of a tracer or arsenic of recent recharge, *C*_2_ is the concentration of a tracer or arsenic of native, intermediate groundwater, *C*_3_ is the concentration of a tracer or arsenic of native, deep groundwater, *f*_1_ is the fraction of recent recharge, *f*_2_ is the fraction of native, intermediate groundwater; (1 − *f*_3_ − *f*_1_), *f*_3_ is the fraction of native, deep groundwater.

Tracer concentrations of each fraction were calculated by convolution of the tracer input history with the dispersion model (DM) (Equation 2; Maloszewski and Zuber 1982; Jurgens et al. 2012). Because the screen length is long (252 m) and much of the groundwater in the study area was predominantly old, it was expected that measured ^14^C concentrations represent the mean concentration of many parcels of water entering the well encompassing a broad set of travel times. For this reason, dispersion models were used so that dispersion processes affecting the age distribution could be simulated in the mixture.
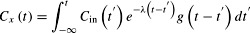
(3) where C*_x_*(*t*) is the outlet tracer concentration for one of the fractions in the ternary mixing model, *C*_in_(*t*′) is the concentration of a tracer at an inlet at time *t*′, *t* is the sample date, *t*′ is the date at which a water parcel entered the system, λ is the decay constant, fractional loss per unit of time, and *t – t*′ is the age of the water parcel. *g*(*t* − *t* ′) is the transit time density function of the dispersion model.

The ternary mixing model (Equation 2) has nine parameters: two independent mixing fractions (*f*_1_ and *f*_3_), a mean age and dispersion parameter for each dispersion model, and an unsaturated zone travel time. Only three tracers were used to calibrate the mixing model—^3^H, CFC-113, and ^14^C. Consequently, several parameters were fixed or assumed while other parameters were free in order to find solutions to the model. The unsaturated zone travel time, along with the mean age and dispersion parameters (ratio of dispersive mixing to advection) of the intermediate and deep fractions, were fixed to values determined in Bexfield et al. (2012), as discussed in more detail in the Mixing Model Development section of this paper. Model solutions were found by calculating the total relative error incrementally over a range of possible values for each independent parameter. The total relative error is the absolute value of the difference between measured and modeled concentrations divided by the measured concentration; this value distributes the error evenly among the tracers included in the model (that is, the magnitude of concentration is negated). The independent parameters were the mean age and dispersion parameter of the young fraction (*C*_1_) and the mixing fractions of the young and deep fraction (*f*_1_ and *f*_3_). The intermediate fraction, *f*_2_, was calculated from the young and deep fractions. This method results in several thousand model calculations over the parameter space. Model solutions with a total relative error less than 10% were used to define the range of acceptable parameter values and model solutions. This subset typically consisted of about 1500 solutions.

Mixtures estimated using ^14^C data require consideration of concentrations of dissolved inorganic carbon (DIC) because the total ^14^C content in a water sample depends on the concentration of DIC in each component of the mixture. However, Bexfield et al. (2012) found that median DIC concentration (as measured by alkalinity titration) were similar in groundwater from shallow, intermediate, and deep parts of the aquifer (97, 114, 106 mg/L as calcium carbonate, respectively). Consequently, the effect of DIC on ^14^C concentrations likely is not significant in this system and therefore was not considered in model calculations.

## Results and Discussion

### Concentrations of Age Tracers and Arsenic

The concentrations of ^3^H, CFC-113, ^14^C, and arsenic in water from the well vary according to the time of year (or pumping season) when the well is sampled (Table 1). ^3^H, CFC-113, and ^14^C concentrations were highest in the June 2007 and May 2009 sample sets, while the DD (December 2007) and November 2008 samples had the lowest concentrations. The number of VOCs detected in a sample were highest in the June 2007 and May 2009 sample sets, while arsenic was highest in the DD and November 2008 samples. The chemical variation of water produced from the ASW reflects the response of the well to seasonal pumping cycles and subsequent changes in hydraulic head in the aquifer.

**Table:** Measured Concentrations of Arsenic and Tracers of Groundwater Age, Number of VOCs, and Calibrated Carbon-14 Ages from a PSW in Albuquerque, New Mexico

Sample Name	Sample Date	Sample Time	Number of Volatile Organic Compounds	Tritium (TU)	Tritium Uncertainty, 1-Sigma (TU)	Calculated Atmospheric Mixing Ratio of CFC-113 (pptv)	CFC-113 Uncertainty from Recharge Temp and Elevation (pptv)	Corrected ^14^C (percent modern Carbon)	^14^C Error (percent modern Carbon)	Conventional Radiocarbon Age (years BP)	Radiocarbon Age Error, 1-sigma (years)	Calibrated ^14^C Piston-Flow Age^1^, with Range in Parentheses (years BP)	Arsenic (mg/L)
June 2007	June 7, 2007	1400	1	2.35	0.09	5.4	0.5	44	0.3	6582	63	7480 (7340–7580)	11.9
DD, 115	December 4, 2007	1200	0	0.54	0.09	0.1	0.0	22	0.2	12,110	25	13,950 (13,810–14,090)	18.1
DD, 133	December 4, 2007	1400	0	0.64	0.09	0.5	0.0	22	0.2	12,286	30	14,160 (13,980–14,830)	18.3
DD, 151	December 4, 2007	1600	0	0.77	0.09	10.5	0.9	25	0.2	11,006	30	12,870 (12,700–13,070)	16.8
DD, 176	December 4, 2007	1700	0	0.14	0.09	3.6	0.3	12	0.2	17,211	62	20,410 (20,160–20,980)	17.8
DD, 241	December 5, 2007	1300	0	0.06	0.09	0.3	0.0	10	0.2	18,695	83	22,310 (22,070–22,510)	23.3
DD, WH	December 5, 2007	1500	0	0.70	0.09	0.3	0.0	24	0.2	11,244	49	13,150 (12,960–13,290)	18.4
November 2008	November 10, 2008	930	1	0.96	0.09	0.6	0.1	31	0.2	9298	48	10,500 (10,300–10,650)	16.5
09TS1	May 15, 2009	1900	2	1.93	0.09	0.8	0.1	42	0.1	7020	44	7860 (7750–7950)	12.8
09TS2	May 16, 2009	810	3	2.03	0.09	1.2	0.1	42	0.1	7056	44	7860 (7750–7950)	12.2
09TS3	May 16, 2009	1840	2	1.82	0.09	1.7	0.1	42	0.1	7056	44	7860 (7750–7950)	12.3
09TS4	May 17, 2009	800	2	2.01	0.09	7.0	0.6	42	0.1	7056	44	7860 (7750–7950)	12.1
09TS5	May 17, 2009	1830	2	1.80	0.09	3.1	0.3	42	0.1	7056	43	7860 (7750–7950)	12.2
09TS6	May 18, 2009	745	1	2.06	0.09	1.2	0.1	41	0.1	7075	44	7900 (7800–7980)	12.1

Note: mbls, meters below land surface; ug/L, micrograms per liter; TU, tritium units; pptv, parts per trillion by volume; BP, before present (1950).

1 Table 1Calibrated carbon-14 age is the age at the median probability distribution for 2-sigma error; see text for details.

During prolonged periods of nonpumping, water-level gradients and flowmeter results show that upward gradients in the well allow deep groundwater to migrate upward through the wellbore (or annular material) and move out into the aquifer at intermediate depths (approximately 110 to 150 mbls; Figure 3). This process results in the storage of deep groundwater at intermediate depths until pumping resumes (see Bexfield and Jurgens, 2014). Consequently, when the well is sampled after long periods of nonpumping, concentrations of ^3^H, CFC-113, and ^14^C and the number of VOCs were lower and arsenic concentrations were higher because the well received groundwater from the deep part of the aquifer plus deep groundwater stored at intermediate depths while the well was idle. During the summer when water demand is high and pumping is more continuous, the influence of deep groundwater stored at intermediate depths is minimized such that the chemistry of water from the well is more representative of groundwater contributed from intermediate depths in addition to the groundwater contributed from the deep part of the aquifer.

Arsenic was above the USEPA MCL of 10 µg/L in all samples, although water from the well was blended and (or) treated to meet drinking-water standards prior to delivery to consumers. The highest arsenic concentration, 23 µg/L, was found in the deepest DD sample collected at 241 mbls and indicates the source of high arsenic to the well was from the deep part of the aquifer. DD sampling also indicates that the majority of young (less than 60 years) water enters the well between 151 and 176 mbls because the highest concentrations of ^3^H, CFC-113, and ^14^C were detected in the DD, 151 sample. Lower concentrations of these tracers in samples above this depth indicate that the water entering the well at shallower depths contains lower concentrations, thereby diluting the concentrations from below.

Conventional radiocarbon ages, in years BP relative to 1950, were used to calculate calibrated ^14^C piston-flow ages by using the program CALIB (Stuiver and Reimer 1993) and the 2009 international calibration curve, IntCal09 (Reimer et al. 2009). Because of paleoatmospheric fluctuations in ^14^C levels, the radiocarbon age (along with the measurement error) can correspond to multiple calendar ages. Therefore, radiocarbon ages typically have a range of calendar ages. The reported ^14^C piston-flow age is the age at the median probability (assuming that the age range is normally distributed about the radiocarbon age) of the distribution of the range of ages given in parentheses to the right of the piston-flow age (Table 1) (Stuiver and Reimer 1993). Calibrated ^14^C ages for samples from the ASW ranged from 7480 to over 22,300 years and differences in the ages reflect the ^14^C patterns discussed above.

### Mixing Model Development

**Figure 4:**
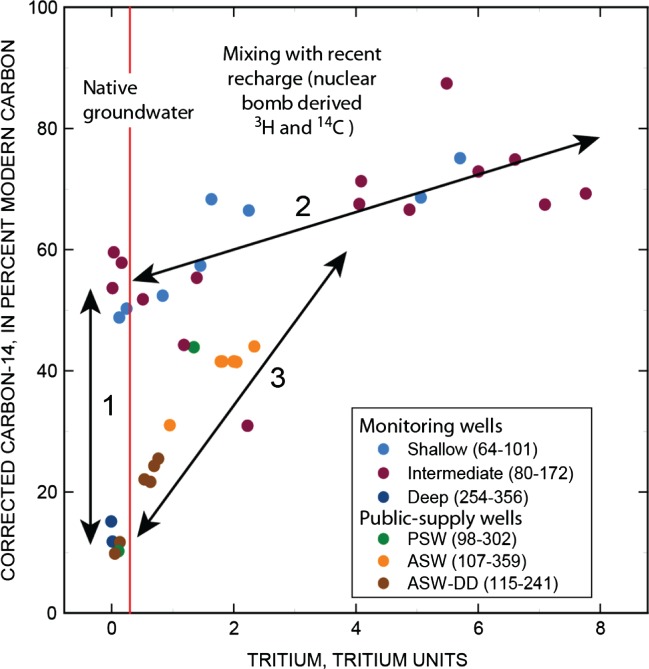
Carbon-14 and tritium concentrations in groundwater samples collected from monitoring wells screened at shallow, intermediate and deep depths, and from the Albuquerque supply well (ASW) and other PSWs in the study area. Depth-dependent samples (ASW-DD) were collected from within the pumping supply well. The range of depths (from land surface) covered by the screened intervals or sample point is given in parentheses. Red vertical line shows separation between native groundwater that is primarily unaffected by recent recharge and old groundwater that has been affected by recent recharge. Numbers and arrows represent mixing between groundwater: (1) mixing that would occur in a PSW producing only native groundwater from deep and intermediate depths, (2) Mixing of recent recharge with native groundwater at shallow and intermediate depths, and (3) Mixing in a PSW of native groundwater at deep depths, native groundwater at intermediate depths, and recent recharge that has migrated to intermediate depths (Bexfield et al. 2012).

The ASW has a screen interval from 107 to 359 mbls and captures groundwater from intermediate and deep parts of the aquifer (Figure 3). Tracer concentrations and wellbore flow data from the pumping well and previously published groundwater chemistry data (Figure 4) indicate that three isotopically distinct waters in the alluvial aquifer of the MRGB enter and mix in the PSW. Although the PSW is screened across only the intermediate and deep depth zones, groundwater at intermediate depths in the aquifer was shown to be a mixture of young (between 18 and 25 years) and old (about 6500 years old), native groundwater. In addition, storage of deep groundwater at intermediate depths in the vicinity of the ASW results in a ternary mixture of water entering the well at intermediate depths. For modeling purposes, the fraction of deep groundwater entering the well from the deep depth zone and the fraction of deep groundwater stored at intermediate depths was treated as a single fraction that could be separated after model calibration. Consequently, the three components in the mixing model include: (1) recently recharged groundwater (less than 60 years) at intermediate depths (between 151 and 176 mbls), (2) relatively old (approximately 6500 years old), native (^3^H free) groundwater at intermediate depths, and (3) very old (approximately 21,000 years old), native deep groundwater (Figures 3 and 4). It was important to distinguish between the two fractions of old (native) groundwater in the model because only the oldest groundwater contributes arsenic to the well at concentrations exceeding the USEPA MCL of 10 µg/L (Bexfield et al. 2012).

Bexfield et al. (2012) found that groundwater at shallow and intermediate depths within the study area often contains water that was recharged within the last 60 years. This groundwater contains ^3^H and ^14^C derived from the aboveground nuclear weapons testing (Figure 4). Native groundwater that is relatively free of recent recharge contains ^3^H concentrations of less than 0.3 TU, the smallest detectable concentration expected in groundwater from the decay of the ^3^H precipitation record to the year 2008.

The first component, *f*_1_, of the ternary mixing model equation (Equation 2) represents the fraction of young, recently recharged groundwater from intermediate depths. This fraction was allowed to vary from 0.05 to 0.15 for summer samples and from 0.01 to 0.1 for winter samples during the search for model solutions. These ranges were based on binary dispersion model and inverse geochemical model results from Bexfield et al. (2012) who found the young fraction in summer samples was about 11% and about 3% to 5% in winter samples. The oldest fraction, *f*_3_, should account for about 35% of the flow contributed to the well based on wellbore flow data (Figure 2); however, this fraction was allowed to vary from 0.3 to 0.9 to account for contributions of flow from additional fractions of deep groundwater stored at intermediate depths and (or) to account for wellbore flow measurement uncertainty. Wellbore flow data also indicate that the total intermediate fraction (native, intermediate groundwater plus young groundwater) should be about 65% of the flow to the well, assuming negligible storage of deep groundwater at intermediate depths.

The concentration of the youngest fraction, *C*_1_, was simulated with a dispersion model with a mean age that was tested over the range from 18 to 25 years, which is based on the range of mean ages of young groundwater in shallow and intermediate depth groundwater (Bexfield et al. 2012). The dispersion parameter was varied from 0.01 to 0.05, because plots of tracer concentrations for different mean ages showed that the combination of tracers (^3^H, CFC-113, and ^14^C) in groundwater could only be explained using dispersion parameters in this range. Larger values of the dispersion parameter increase the dispersion of tracer concentrations in groundwater (i.e., broader and shorter peaks) and lead to greater discordance between the measured tracers. The range of tested values indicate that tracer concentrations in groundwater are not dominated by either advection or dispersive mixing although they tend to be more reflective of advection processes. It is important to note that Maloszewski and Zuber (1996) indicate that the dispersion parameter in the DM may have little connection to actual dispersivities measured at the field scale and more generally controls the distribution of transit times in the model, particularly when determined from inverse calibration of the DM to tracer data.

The concentration of native, intermediate groundwater, *C*_2_, was computed using a dispersion model with a mean age of 6500 years and a dispersion parameter of 0.01. The mean age and dispersion parameter for this DM were fixed parameters in the model and were estimated by Bexfield et al. (2012) using a sample collected during the summer from a nearby monitoring well screened at intermediate depths (see well FP1MS in Bexfield et al. 2012). The concentration of the oldest fraction, *C*_3_, was assumed to contribute water that was 21,120 years old, which was the age obtained by Bexfield et al. (2012) from modeling the DD sample collected at 176 mbls with a dispersion model using a dispersion parameter of 0.01. The dispersion parameter used in the intermediate and deep model components were chosen arbitrarily to simulate some dispersion of tracer concentrations in groundwater rather than a purely advective (piston-flow) concentration, which is unrealistic. The mean ages and dispersion parameters used in the intermediate and deep models were fixed parameters. The mean ages used for intermediate and deep groundwater were representative of nearby intermediate and deep groundwater which had median mean ages of 6250 and 18,100 years, respectively.

Inverse calibration of LPMs with tracer data from monitoring wells in the study area by Bexfield et al. (2012) revealed a consistent pattern of travel time delays of tracers in groundwater. On the basis of that finding, all DMs included a travel time delay through the unsaturated zone of 15 years as reported by Bexfield et al. (2012)—a travel time that is consistent with the thick unsaturated zone (up to about 75 m) in the area.

### Mixing Model Results

Final mixing model solutions for the four water samples from the ASW are reported in Table 2. The solution results correspond to the absolute minimum error for each sample for all mixing fractions and parameter sets tested by the ternary model, although a number of models with slightly different mixing fractions and ages of water were identified that could be consistent with measured tracer concentrations in the ASW samples and samples from the monitoring wells in the area. Alternative models generated from the tested parameter set models were considered acceptable when the total error was less than 10% for the samples in June 2007 and 09TS4 and less than 5% for the depth-dependent wellhead sample (DD, WH) and the November 2008 sample. The lower total error criteria for the latter two samples was used because the CFC-113 concentrations in those samples were too low and uncertain to be used as a constraint in the models. The acceptance criteria were arbitrary values chosen to define a number of possible model solutions that can be used to characterize the variability of model solutions given the uncertainty in measured tracer concentrations. A more rigorous uncertainty analysis of model calculations would account for the measurement uncertainty in the solution finding technique but that effort was beyond the scope of this paper.

**Table:** Ternary Mixing Model Results

Sample Name	Number of Acceptable Models	Mean Age of Young Fraction (years)	Dispersion Parameter of Young Fraction	Fraction of Young Water (%)	Fraction of Native Intermediate (%)	Fraction of Deep Water (%)	Total Relative Error (%)	Total Fraction of Intermediate Water (%)	Excess Proportion of Old gw Stored in Intermediate Zsone^1^ (%)	Modeled Arsenic (µg/L)^2^
June 2007	1816	20.9	0.05	10	51	39	0.52	61	4	12.0
DD, WH	795	20.9	0.03	3.2	23	74	0.09	26	39	18.3
November 2008	1664	20.1	0.02	6.4	30	64	0.12	36	29	16.5
09TS4 (May 2009)	2036	20.9	0.05	11.4	40	49	1.60	51	14	13.8

Note: All model results assume an unsaturated zone travel time of 15 years, an intermediate and deep mean age of 6500 and 21,120 years, respectively, and 0.01 was used for the intermediate and deep model dispersion parameter values.

a Table 1Assumes water entering well at intermediate depths comprises 65% of total flow to well (based on flow log).

b Table 2Estimated by mixing 23 and 5 µg/L (value from adjacent monitoring well) of old and intermediate gw.

The number of acceptable models ranged from about 800 to 2000 (Table 2). For each model set, a one-sigma standard deviation of all model solutions was calculated for each model parameter tested. The one-sigma error for mean age of the young fraction was less than 1 year and for the dispersion parameter was less than 0.015 for all model calculations. The one-sigma error for estimated fractions of deep and young groundwater in the models was less than 4% and 1%, respectively. Increasing the acceptance criteria by 5% only increased the error for the fractions of deep and young groundwater by 1%.

**Figure 5:**
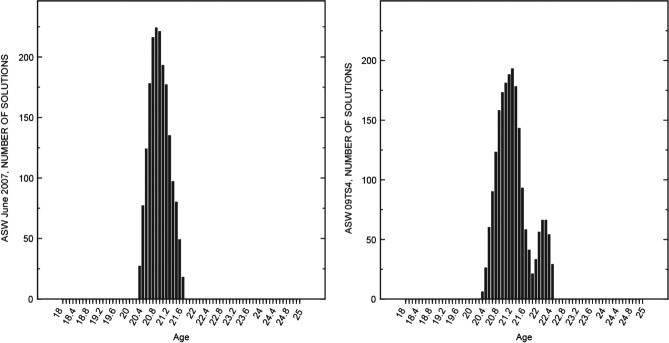
Number of model calculations with less than 10% total error for various simulated ages of recent recharge (*f*_1_) for samples collected from the PSW in June 2007 and May 2009 (09TS4).

The age of the young fraction for the summer samples was between 20 and 23 years in all model calculations having a total error less than 10% (Figure 5), although the tested range included ages between 18 and 25 years. On the basis of these results, the tested age range of the young fraction in models for winter samples was restricted to between 20 and 23 years because the CFC data were not useful for further constraining the age range of the young fraction in the samples. The age of the young fraction in the final models was about 20 to 21 years (Table 2). If the travel time through the unsaturated zone estimated by Bexfield et al. (2012) is considered, then the total elapsed time since water first entered the ground and traveled to the well was 35 or 36 years.

**Figure 6:**
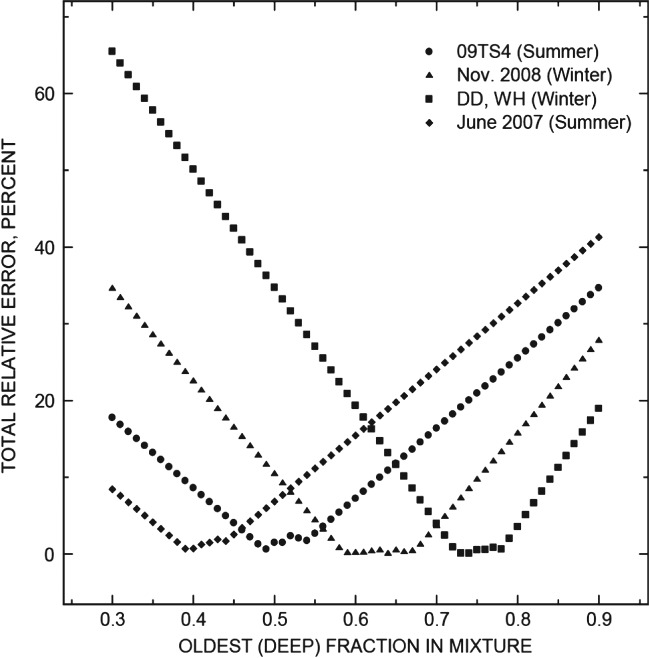
Solution errors for various fractions of the oldest, deep groundwater in age mixture models.

The ternary models for each sample were calculated over a range of fractions of deep groundwater (Figure 6). Consequently, there were a number of acceptable solutions over this range as mentioned above, given the uncertainty of measurement error. If only model calculations having a total error of less than 1% were considered, then the number of potential solutions generally occur within a small window of about 0.05 fractional increments of deep groundwater, although the 2008 sample has a relatively large window of about 0.1 fractional increments (Figure 6). For this study, only the model with the minimum error over the entire interval range is presented as the best model for a sample and is listed in Table 2.

Model results show that the fraction of deep, very old groundwater (more than 21,000 years) was higher in winter samples than in summer samples, while the fraction of young groundwater contributed to the ASW was larger for the summer samples than for the winter samples (Table 2). Fractions of deep groundwater ranged from 39% to 74%, while the young fraction ranged from 3% to 11%. The fraction of deep groundwater for the June 2007 model sample was 39%, which is close to the fraction estimated from flowmeter measurements of about 35%, although the deep fractions modeled for other samples were substantially more than 35%. The fraction of young groundwater ranged between 10% and 11% during the summer, but may only comprise about 6% during the winter. These results are similar to results from a groundwater flow and transport model of the area, which found that the fraction of young (post-1950s) groundwater in the well comprises about 2% and 8% during the winter and summer seasons, respectively (Heywood written communication, 2012).

The variability of fractions of young and old groundwater reflect the hydraulic responses of the aquifer to seasonal patterns of intense pumping during the summer and little to no pumping during the winter (see Bexfield and Jurgens, 2014). As stated above, water-level gradients and flowmeter results show that upward gradients exist in the well under ambient conditions, allowing deep groundwater to migrate upward through the wellbore (or annular material) and move out into the aquifer at intermediate depths (approximately 110 to 150 mbls). This process results in the storage of deep groundwater at intermediate depths until pumping resumes (see Bexfield and Jurgens, 2014). Consequently, when pumping resumes, the well receives groundwater from the deep part of the aquifer and deep groundwater stored at intermediate depths. The amount of deep groundwater stored at intermediate depths is a function of the amount of time the well is off and how frequently the well is pumped. The amount of stored groundwater should decrease as the amount of time the well is pumped increases. Likewise, the amount of total intermediate water entering the well should increase as the pumping period increases.

The fraction of young water is a component of the water that enters the well at intermediate depths (110 to 215 mbls). If storage of deep groundwater at intermediate depths did not occur, then the combined fractions of young and native, intermediate depth groundwater (total intermediate fraction) should be similar to the contribution of groundwater at intermediate depths measured by the flowmeter, which was about 65%. However, the models suggest the total intermediate depth fraction of water produced by the well ranges from as much as 61% for the June 2007 sample to as little as 26% for the winter, depth-dependent sample. Therefore, the difference between the flowmeter measured contribution and the estimated total fraction of intermediate groundwater can provide an estimate of the amount of deep groundwater stored at intermediate depths under different pumping conditions.

The total fraction of old groundwater stored at intermediate depths was lowest in June 2007 and highest in December 2007 (Table 2). Specifically, the fraction ranged from a low of 4% in June 2007 (summer) to a high of 39% in December 2007 (winter). These are the latest summer and winter samples, respectively, and it is not surprising that the lowest fraction of old, stored groundwater would be observed when the well was frequently pumped (summer sample) and the highest fraction would be associated with an extended period of nonoperation (the well was offline for several weeks before the DD sampling was conducted).

### Estimated Arsenic Concentrations

Arsenic concentrations were highest in samples collected from the ASW during the winter months (Table 1), when pumping was at a minimum and the storage of deep, high-arsenic groundwater at intermediate depths was greatest. The ternary models and the estimated fractions of water that contribute to the mixture were used to test the ability of the models to estimate concentrations of arsenic in the mixtures.

Arsenic concentrations were estimated from the fractions of young, intermediate, and deep groundwater entering the well. The deep fraction was assigned a value of 23 µg/L, which is equal to the arsenic concentration entering the bottom of the well (Table 1; Figure 3), while the intermediate and young fractions were assigned a concentration of 5 µg/L, which was estimated from arsenic values from a nearby monitoring well screened at intermediate depths (see FP1MS in Bexfield et al. 2012). The arsenic values were calculated from the fractions multiplied by their assigned concentrations and summed to yield a simulated arsenic concentration for the mixture (Table 2).

All but one of the samples showed good agreement between the simulated and actual arsenic concentrations for the well. Three of the four simulated arsenic concentrations were within 0.1 µg/L of the actual value, while the May 2009 sample was overestimated by almost 2 µg/L. This discrepancy suggests the fraction of old groundwater in the sample was overestimated by the model by about 10% or that the concentrations assigned to the model do not reflect the actual concentrations in the mixture. Since the ^3^H and ^14^C values are consistent with a slightly larger fraction of old groundwater in comparison to the sample in June 2007, the discrepancy between modeled and measured arsenic concentrations could be due to concentration differences in the fractions that make up the mixture at that particular time of sampling.

From these mixing models, the arsenic concentration for the well under normal conditions, where no deep groundwater is stored at intermediate depths, was estimated to range between 9.6 and 10.2 µg/L. These values are similar to historical summer samples from the ASW (when deep groundwater storage should have been minimal) that generally ranged from 10 to 11 µg/L (25th and 75th percentile). The models determined for this paper only estimate arsenic concentrations for recent aquifer conditions and may not reflect past conditions when pumping was lower and water levels were higher.

## Summary and Conclusions

The age distribution of groundwater reaching a PSW in Albuquerque, New Mexico was estimated from environmental tracer concentrations of ^3^H, CFC-113, and ^14^C using a three component mixing model of relatively young groundwater (less than 60 years old), old (native), intermediate groundwater (about 6500 years old), and very old (native), deep groundwater (more than 21,000 years old). The oldest fraction of water reaching the well varies with pumping season and was highest during the winter when pumping was at a minimum. The fraction of deep groundwater ranged from a low of 39% in June 2007 to a high of 74% in December 2007. Although the young fraction also varied with pumping season, the fraction of young water was highest during the summer (11.4%), when pumping was at a maximum, and lowest during the winter (3.2%).

The large fractions of deep groundwater in the mixtures result primarily from the contribution of deep groundwater at deep depths and from the storage of deep groundwater at intermediate depths. Under nonpumping conditions, the natural upward hydraulic gradients promote the movement of deep groundwater through the borehole or annular space and out of the borehole and into the aquifer at intermediate depths, where it is stored until pumping resumes. Although the fraction of groundwater entering the well at deep depths comprises approximately 35% according to the flowmeter log, the well often receives a larger fraction of groundwater derived from deep depths during pumping because of deep groundwater stored at intermediate depths. The excess deep groundwater that is stored at intermediate depths also dilutes and minimizes the fraction of young groundwater contributed to the well during these periods, which is primarily during the winter months when water demand is low.

Because deep groundwater water in the area contains high concentrations of arsenic, larger fractions of deep groundwater during the winter cause arsenic concentrations to be greatest. Deep groundwater from the pumping ASW had an arsenic concentration of 23 µg/L, and monitoring wells in the area that are screened at deep depths had arsenic concentrations as high as 35 µg/L (Bexfield et al. 2012). Arsenic concentrations were computed using the mixing model, and close agreement was found with measured arsenic levels in the well in 3 of 4 samples. Arsenic was above the USEPA MCL of 10 µg/L in all of the samples.

Arsenic concentrations are the most important health concern for the ASW and other PSWs in Albuquerque, New Mexico (see Bexfield and Jurgens, 2014). The Albuquerque Bernalillo County Water Utility Authority has had to blend groundwater from affected PSWs like this one with lower-arsenic groundwater or surface water before the water is delivered to consumers. Because arsenic is associated with the deep fraction of groundwater entering the well, altering the well screen might be an appropriate solution to reduce arsenic concentrations in wells. If the screened portion contributing arsenic in the ASW was sealed, the amount of flow contributed to the well would be reduced by about 35% according to the flowmeter log. The drop in production may be acceptable, but this change also increases the vulnerability of the well to anthropogenic contaminants in recently recharged water because the fraction of young groundwater in the mixture would no longer be diluted by deep groundwater. Estimates of the fraction of young groundwater in the mixture, however, only increase marginally to 15.4% and 17.5% for the June 2007 and May 2009 samples. Thus, the removal of deep groundwater may only cause the fraction of young groundwater to increase to about 20%; although it should be noted that long-term pumping could cause the fraction of young groundwater to increase as persistent and increasing downward gradients promote larger volumes of young groundwater to migrate to deeper depths.

Finally, the mixing model developed in this paper provides a plausible solution to the age mixture of a PSW in Albuquerque, New Mexico that was sampled as part of the NAWQA program's TANC topical study (Eberts et al. 2005). A description of the age distribution provides value beyond the ability to estimate arsenic concentrations in a well. The age distribution can be used to understand the intrinsic susceptibility of a well or the response of a well to a nonpoint source contaminant whose history may be anthropogenic in origin rather than natural. The collection of age tracer data has become more commonplace in the last decade and these efforts can provide a more complete picture of the distribution of age and the vulnerability of the aquifer as a whole.
